# Recent Advances in Antivirals for Japanese Encephalitis Virus

**DOI:** 10.3390/v15051033

**Published:** 2023-04-23

**Authors:** Yongzhe Zhu, Shenglin Chen, Qilin Lurong, Zhongtian Qi

**Affiliations:** 1 Department of Microbiology, Faculty of Naval Medicine, Naval Medical University, Shanghai 200433, China; zhuyz@smmu.edu.cn; 2 Department of Clinic Laboratory Diagnostics, General Hospital of Tibet Military Area Command of PLA, Lhasa 850007, China; chenshenglin3534@163.com; 3 Department of Geriatrics, General Hospital of Tibet Military Area Command of PLA, Lhasa 850007, China

**Keywords:** Japanese encephalitis virus, viral components, host factor, innate immune response, inflammatory response, antivirals

## Abstract

Culex mosquitoes are the primary vectors of the Japanese encephalitis virus (JEV). Since its discovery in 1935, Japanese encephalitis (JE), caused by JEV, has posed a significant threat to human health. Despite the widespread implementation of several JEV vaccines, the transmission chain of JEV in the natural ecosystem has not changed, and the vector of transmission cannot be eradicated. Therefore, JEV is still the focus of attention for flaviviruses. At present, there is no clinically specific drug for JE treatment. JEV infection is a complex interaction between the virus and the host cell, which is the focus of drug design and development. An overview of antivirals that target JEV elements and host factors is presented in this review. In addition, drugs that balance antiviral effects and host protection by regulating innate immunity, inflammation, apoptosis, or necrosis are reviewed to treat JE effectively.

## 1. Introduction

Japanese encephalitis virus (JEV) is a single-stranded plus-stranded RNA virus belonging to the genus *Flavivirus* of the family *Flaviridae* [1]. Based on the nucleotide sequence of the envelope (E) gene, JEV can be divided into five genotypes: GI, GII, GIII, GIV, and GV [2]. GI type is the predominant endemic strain throughout Asia and the Indo-Pacific region [3]. As an arthropod-borne virus, JEV is transmitted by the bites of Culex species mosquitoes, particularly Culex tritaeniorhynchus [4]. Domestic pigs and water birds have been recognized as the two most important JEV-amplifying hosts, which have high enough levels of blood viremia to enable transmission to mosquitoes [5]. Humans and horses are considered to be dead-end hosts [6]. The prevalence of JEV shows specific distribution characteristics on the geographical scale, seasonal scale, climate scale, and population scale [7]. In temperate and subtropical regions, populations in agricultural areas are the primary populations exposed to JEV with the onset of the rainy season, among which, unvaccinated populations and children are at high risk of JEV infection. 

The main endemic areas of JEV include Eastern, Southern, and Southeastern Asia, the Western Pacific coast, central and eastern Australia, etc. [8]. According to data released by the World Health Organization in 2011 [9], there are approximately ~68,000 cases of JEV infection each year, including at least 13,000 deaths. Most JE cases are asymptomatic or mild and cause fever and headache; a case fatality rate of ~30% can be observed in those with encephalitis, and permanent neurological or psychiatric sequelae can occur in approximately 30–50% of cases [10]. Several vaccines have been applied in order to prevent the effects of JEV infection, including the attenuated vaccine SAl4-14-2, which has been applied in many countries, inactivated mouse brain-derived vaccines, inactivated Vero cell culture vaccines, and attenuated chimeric vaccines [11]. As such, the incidence of JEV has decreased significantly globally, but vaccination rates remain low in less developed areas. Combined with climate change and the zoonotic cycle of JEV, it is still circulating in some areas. In 2022, 30 confirmed JE cases and 6 deaths were reported in Australia [12]. Due to the convenience and cost-effectiveness of drug therapy, the development of anti-JEV-specific drugs remains a necessary option. This article focuses mainly on a review of the recent developments in research and the mechanisms of antiviral drugs for JEV.

## 2. Structural and Nonstructural Components of JEV

JEV’s genomic RNA size is about 11 kb, with a methylated cap structure at the 5′ terminus and no poly (A) at the 3′ terminus. A single open-reading-frame (ORF) is found between the 5′- and 3′- untranslated regions (UTRs); these encode three structural proteins, including capsid(C), precursor membrane (PrM), and envelope, and seven nonstructural (NS) proteins, including NS1, NS2A, NS2B, NS3, NS4A, NS4B and NS5 [13]. Structural and nonstructural proteins are essential targets for antiviral drug design.

### 2.1. Structural Proteins

The JEV C protein, with a molecular weight of 11 kDa, can pack viral RNA and is the core component of the nucleocapsid. Each monomer molecule of the C protein dimer has four α-helix structures. C protein dimerization is essential for its association with viral RNA [14,15]. 

The E proteins form the outer shell and are anchored to the lipid bilayer envelope through their transmembrane helices and the M protein. The E protein plays a role in receptor binding and membrane fusion and belongs to the classII fusion glycoprotein and contains five domains, among which DI, DII, and DIII are the ectodomains of the virus. The DI domain connects the DII and DIII domains and participates in E protein conformational changes and stability. The DII domain contains fusion peptides and has the function of viral membrane fusion. The fusion peptide of one E monomer is buried between DI and DIII of the adjacent monomer within a dimer. DIII, containing linear antigenic epitopes, is regarded as an antigen [16]. The prM protein might function as a chaperone for the folding and assembly of the E protein [17]. In the trans-Golgi network, furin or furin-like protease cleave the prM protein to produce M protein. The outer surface of mature JEV virions comprises 180 copies of the E and M proteins. Three E-M-M-E heterodimers lying parallel to each other form a raft, and 30 such rafts cover the viral surface [18].

### 2.2. Nonstructural Proteins

JEV nonstructural proteins are essential in viral genome replication, protein translation, and viral particle assembly, and they regulate innate immunity. NS1 is a glycoprotein that plays a role in the replication process of viruses [19]. NS2A is a small hydrophobic transmembrane protein with no enzymatic activity that plays a role in JEV replication and assembly [20]. NS2B is also an integral membrane protein as a cofactor of NS3 and binds to NS3 to perform the NS2B/NS3 protease function [21]. NS3 is a multifunctional enzyme with three domains. The N-terminal of NS3 contains the protease domain, which exhibits serine protease activity. With the cofactor NS2B, NS3 is required to process polyproteins into individual NS proteins. The C-terminal of NS3 has a RNA helicase/NTPase domain and RNA triphosphatase, which are required for unwinding the double-stranded RNA during viral RNA synthesis; this is the first step required for RNA capping by NS5 [22,23]. Due to its crucial enzyme function, NS3 is a key target for the development of anti-JEV drugs [24]. NS4A and NS4B are transmembrane proteins that play a role in viral replication and the regulation of the host immune response [25,26].

NS5 is the most highly conserved and largest nonstructural protein. Its N-terminal contains an S-adenosyl methyltransferase (SAM) (MTase) domain structure and its C-terminal contains an RNA-dependent RNA polymerase (RdRp) domain. MTase has seven β folds, surrounded by four α helices, that can cap viral mRNA. RdRp has RNA-dependent RNA polymerase activity and plays a significant role in viral RNA replication [27,28,29]. In addition to its above function as an enzyme, NS5 can also antagonize the interferon (IFN)-mediated innate immune response through its interaction with hSTAT2 [30]. Due to its highly conserved nature in flaviviruses and its essential role in promoting viral infection, NS5 is a preferred target for anti-JEV drug design [24].

## 3. Lifecycle and Pathogenesis of JEV Infection

The lifecycle of JEV can be divided into entry, RNA replication, viral protein translation, and viral particle assembly and release. An investigation into which stage of the virus lifecycle antiviral drugs act on will pave the way for revealing the specific target of drug action. The replication cycle of JEV is similar to that of other flaviviruses [18,31,32]. Firstly, the E protein binds to functional receptors and adhesion receptors to initiate the endosome-dependent endocytosis pathway. As the virus undergoes membrane fusion with the endosome, the viral genomic RNA is released into the host cytoplasm, initiating the replication journey of JEV. After the synthesis of the viral RNA and proteins, the newly synthesized viral RNA and structural proteins (C, prM, and E) are transferred to the lumen of the endoplasmic reticulum (ER) and assembled into immature infection-defective JEV virus particles. These immature virions are further transported to the trans-Golgi network (TGN), where prM cleavage occurs at specific sites under the action of the cellular serine protease furin and mature infectious virus particles are finally formed. Finally, cellular exocytosis releases mature JEV virions from the host cell. 

JEV is a neurotropic flavivirus; however, its precise route of brain penetration remains unclear. Microglia activation, the innate immune response, the inflammatory response, and neuronal cell death are recognized as essential hallmarks of JE during JEV infection of the central nervous system (CNS). Microglia are considered an essential starting point of inflammatory response when the CNS is triggered by JEV infection [33]. The activation of microglia following JEV infection can release a variety of pro-inflammatory cytokines such as interleukin (IL) and tumor necrosis factor (TNF), triggering a cascade of the inflammatory response, which can prime cell death pathways such as pyroptosis, apoptosis, and necroptosis [34]. The initial line of defense against JEV infection is the host’s innate immune system. Once JEV enters the cell, the viral RNA can be recognized by pattern recognition receptors (PRRs) [35,36], such as Toll-like receptors (TLRs) and retinoic acid-inducible gene I (RIG-I)-like receptors (RLRs). Then, the succeeding signal cascade causes the production of IFNs and pro-inflammatory cytokines such as IL-1 and IL-18, and the released IFNs attach to their receptor, activating a signaling cascade to commence an antiviral state. Although the innate immune response and inflammatory response stimulated by JEV infection are the host cell’s natural defense line against virus infection, an excessive or prolonged inflammatory response can cause harm to the body. To accomplish the dual effect of anti-JEV and host protection, it is crucial to develop drugs that modify the innate immune response and inflammatory response in an optimal manner.

## 4. Antivirals against JEV Infection 

**Figure 1:**
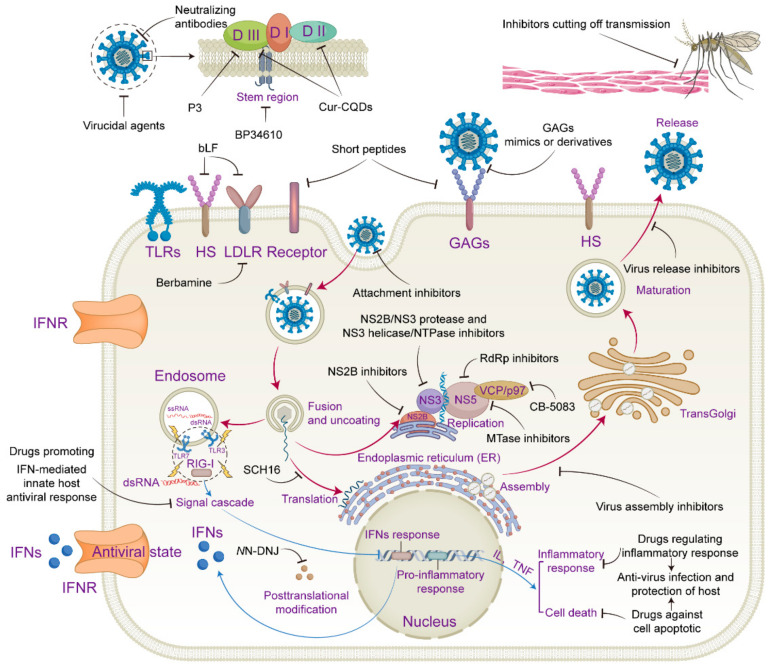
Schematic diagram of antivirals for JEV with different targets. Structural model of JEV virion and E protein are presented extracellularly with extracellular virucidal agents targeting the virion and antivirals targeting the E protein, or separate domains of the E protein; meanwhile, the model of JEV transmission via mosquito bite is also illustrated with drugs that can cut off the transmission route. The lifecycle of JEV and innate immune and inflammatory responses are presented separately with different colored arrow lines, with antivirals targeting viral components or host factors during JEV attachment, entry, RNA synthesis, translation, assembly and release, and with antivirals targeting the modulation of IFN-mediated innate antiviral immunity, inflammatory response and apoptosis.

To date, there are no specific clinical drugs available for JE treatment. Various approaches have been used to develop drugs for JE, which can be summarized in the following three main categories: targeting viral components, targeting host factors, and regulating innate immune and inflammatory responses (Figure 1).

### 4.1. Virucidal Agents Direct Targeting Viral Particles

Some compounds can destroy the envelope integrity of virus particles and play a direct virucidal role. For example, aloe emodin, chrysophanol, indigo, indirubin, and baicalein can cause irreversible damage to JEV virus particles [37,38,39]. Polyoxometalates (POMs) are negatively charged clusters of inorganic substances consisting of oxide anion and transition metal cations. A keggin-type niobium-substituted-heteropolytungstate Cs_2_K_4_Na[SiW_9_Nb_3_O_40_]·H_2_O (named POM-12) could suppress the infection of Dengue virus (DENV), Zika virus (ZIKV) and JEV by disrupting the integrity of these virions [40].

Some biological proteins also show direct virucidal activity. For example, griffithsin (GRFT), a plant-derived broad-spectrum antiviral protein isolated from the red alga *Griffithsia* sp., was proven to have a specific virucidal effect on JEV [41]. 

A recent study found that two lipases, chromobacterium antiviral effector-1 (CbAE-1) and CbAE-2, produced by the *Chromobacterium* sp. Beijing strain isolated from the *Aedes aegypti* midgut, could destroy the integrity of the lipid bilayer structure of the virus envelope and lead to the structural breakdown of virions and RNA release, with a broad-spectrum mosquito-borne virus virucidal activity; it could act on, for example, DENV, ZIKV, JEV, yellow fever virus (YFV) and Sindbis virus (SINV) [42].

Studies of these compounds or proteases with virucidal activity were mainly conducted in the in vitro phase, and their effects on the host cell membrane thus require in-depth evaluation.

### 4.2. Inhibitors Targeting E Proteins

The JEV E protein is an essential viral surface antigen that can bind to the cell membrane surface receptors or adhesion factors, and that mediates the cell entry of JEV. The development of drugs that target the E protein is an effective way to prevent JEV infection. 

Neutralizing antibodies are a classical class of antiviral drugs that bind to antigens on the surface of a pathogenic microorganism, thereby preventing that pathogenic microorganism from adhering to target cell receptors and thus preventing the invasion of the cell. To our knowledge, three E-M-M-E heterodimers lying parallel to each other form a raft on the JEV surface [18]. There are two old monoclonal antibodies (mAbs) named 2F2 and 2H4, firstly reported in the 1980s, that have been demonstrated to bind across three E proteins in a raft and inhibit viral endocytosis and membrane fusion by competitively blocking the binding of the JEV E protein to either the host cell membrane surface receptors or adhesion receptors. The residues Q52, E55, N134, S275, S123, R128, K124, and T205 in the E protein are involved in the interaction of E with both 2F2 and 2H4 fabs. The 100% survival rate of 2F2 and 2H4 against JEV in infected mice and against the undetectable JEV in their brains suggested that 2F2 and 2H4 are promising candidates for the clinical treatment of JEV infection [43]. 

The N-terminal stem region of the JEV E protein is a potential antiviral target. A drug named BP34610 could strongly inhibit the infectivity of DENV2 and JEV in baby hamster kidney-derived (BHK)-21 cells. The target could be the N-terminal stem region of the E protein, of which the S397 site is crucial for the interaction between BP34610 and the DENV E protein. The mutation of S397P causes DENV2 to resist BP34610 [44]. Given the sequence similarity between the JEV and DENV E proteins, it is speculated that the target of BP34610 against JEV infection may also be the E protein, but more details of this must be revealed. 

The resolution of JEV structural proteins provides a fundamental structural biological basis for drug discovery and development. To our knowledge, the DII and DIII of the E protein play essential roles in viral membrane fusion and attachment with host cells [31]. A phage display peptide library was utilized to identify a polypeptide named P3 (amino acid sequence: SENRKVPFYSHS) that significantly inhibited the cell adhesion of JEV by binding to the N-terminal of the DIII of the E protein. Molecular modeling and docking of the E DIII/P3 complex showed that P3 bound to the adjacent areas of the E DIII N-terminal BC-loop and DE-loop via hydrophobic interaction, and that the E protein V357 is the critical amino acid residue for the E protein to bind to P3 [45]. Carbon quantum dots (CQDs) are small-sized carbon nanomaterials with good water dispersion, remarkable optical properties, extreme stability, and high biocompatibility [46]. The guaiacol on the surface of curcumin-CQDs (CUR-CQDs), a nanomedicine synthesized by CQDs and curcumin, can bind to the JEV E DII and DIII via hydrogen bonding and Pi-cation interactions, respectively. In addition, compared to curcumin, Cur-CQDs have higher water solubility and lower cytotoxicity, and can reduce the production of reactive oxygen species (ROS) in cells, making it a promising alternative drug to curcumin [47].

Aside from blocking the E protein via interaction with Van der Waals’ force or via Pi-cation interactions at specific sites, particular drugs may inhibit JEV infection by interacting with the E protein electrostatically. Negatively charged sulfate groups on the glycosaminoglycans (GAGs) can bind to a cluster of positively charged residues on the JEV E glycoprotein [32]. GAGs such as heparan sulfate (HS) are abundant on the surface of various types of cells. Heparin, a drug with a structure similar to HS, could significantly block the JEV and DENV2 infection of BHK-21 cells by competing with HS for virus binding on cell membranes [48]. In vitro studies have shown that HS mimetic PPS, PI-88, and suramin, which have been widely used in clinical practice, all have significant anti-JEV and DENV-2 infection activity in vitro. At the same time, only PI-88 could ameliorate disease outcomes in a JEV- and DENV-2-infected animal model, suggesting that the in vitro anti-flaviviral effectiveness of the HS mimetics did not reliably predict their in vivo therapeutic activity [49]. A derivative of HS, derived from shrimp (*Penaeus brasiliensis*) heads with significantly different sugar chains from heparan, displayed a strong inhibitory effect on JEV infection in vitro [50], although there were some GAG derivatives that exhibited a paradoxical effect on the inhibition of the JEV infection of different cells [51]. However, the unique saccharide sequence in the molecular structure of shrimp HS is considered to be a key site of binding with the surface of the JEV particle, suggesting that the binding of HS/heparan does not depend on simple ionic interaction, but may occur through a specific saccharide sequence. Furthermore, due to the lower anti-thrombin activity of shrimp HS, shrimp HS could be used as a potential anti-JEV drug to replace commercial heparan [50]. 

### 4.3. Inhibitors Targeting NS2B/NS3 Protease

The NS2B/NS3 protease is an important target for the design of anti-JEV drugs [52]. As a cofactor of NS3, NS2B is essential to the function of NS3 serine protease. To date, there have been few reports regarding inhibitors that directly target NS2B. A docking simulation analysis revealed that 4-hydroxypanduratin A, a secondary metabolite of the *Boesenbergia pandurata* Schult (Fingerroot) plant with various pharmacological effects, such as neuroprotective, potent antioxidant, antibacterial and antifungal effects, could interact with the hydrophilic domain of NS2B in JEV via two H-bonds (G80 and D81) with active residues. Hence, 4-Hydroxypanduratin A is a potential JEV NS2B inhibitor that theoretically affects NS2B/NS3 protease activity [53].

Similarly, some inhibitors targeting specific sites of the NS3 protein can also affect the activity of the NS2B/NS3 protease of JEV and have a broad-spectrum inhibitory effect on flaviviruse replication. Three clinically approved drugs, temoporfin, niclosamide, and nitazoxanide, could effectively inhibit the replication of several flaviviruses, such as DENV2, West Nile virus (WNV), JEV, and YFV; all three drugs bind to NS3 in a non-competitive manner, specifically blocking interactions between the viral NS2B cofactor and the NS3 protease domain in vitro. In particular, temoporfin, with the highest level of binding to NS3, effectively protected mice from the lethal threat of ZIKV. Structural docking analysis showed that temoporfin bound to NS3 pockets that hold critical NS2B residues. It is, therefore, worthwhile to investigate further the anti-JEV potential of these three drugs at the molecular and animal levels [54].

Some critical amino acid residues of viral proteins play essential roles in the interactions between drugs and viral proteins. It is crucial to understand the mechanism of drug action and to predict and analyze the characteristics of virus drug resistance by accurately localizing these critical amino acid residues. Erythrosin B, an FDA-approved food additive, was found to block NS2B binding to the NS3 of several flaviviruses, including JEV, by targeting NS3 via a non-competitive mechanism. Based on the docked structure of the erythrosine B-NS3 complex, the mutants of DENV2 NS3 were constructed, and the Y23, I25, F46, L58, and H60 residues of NS3 were found to play a vital role in the interaction between erythrosin B and NS3 using a protein thermal shift assay (PTSA); these amino acid residues were indispensable for the binding of NS3 and NS2B. As a food additive of pregnancy category B approved by the FDA, erythrosin B has been widely used in many countries. Therefore, erythrosin B may have the potential to be applied in broad clinical settings for the management of infections by JEV and other flaviviruses in the future [55]. A structure-based screening of anti-DENV drugs, combined with a cell-based replication assay, found that a non-competitive chemical inhibitor of NS2B/NS3 protease named SK-12 significantly inhibited all four serotypes of DENV replication by targeting the NS2B binding site of NS3 in vitro; it was also found to have a significant inhibitory effect on the JEV infectivity. By analyzing the structural details of SK-12 and NS3 binding, and the NS3 sequence differences between DENV and JEV, it was found that Q27 and H60 are highly conserved in DENV 1-4 but not in JEV. However, the inhibition efficacy of SK-12 on JEV infection was lower than that of DENV, which is due to the difference between DENV and JEV at the 27th amino acid residue of NS3; this was revealed by a mutagenesis assay, wherein NS3 containing Q27 was found to be highly sensitive to SK-12, which is part of the pocket holding the NS2B residue [56]. In this regard, computer drug design provides a theoretical reference for analyzing the molecular mechanisms of drug–viral protein interactions, but the details will need to be validated and confirmed through the use of functional experiments.

Besides the drugs or inhibitors above, several other inhibitors, only investigated in an in silico assay, have been found to target NS2B/NS3 protease; these include andrographolide [57], abscisic acid, and aloe-emodin [58], all of which were found to inhibit the NS3 protease of JEV in an vitro target-based enzymatic assay and exhibited their particular potential to combat JEV infection. However, more detailed experimental studies that focus on these drugs are required in order to reveal their function as specific protease inhibitors of JEV.

### 4.4. Inhibitors Targeting NS3 Helicase/NTPase

Antiviral peptides are a group of important antiviral drugs. Viruses of the *Flaviviridae* family possess a similarly structured NS3 protein with highly conserved Arg-rich motifs of the NTPase/helicase domain. Short peptides that are designed based on these conserved sequences could theoretically competitively inhibit NS3 helicase/NTPase activity. For example, some peptide inhibitors, such as the JEV (amino acid 1962–1975 of JEV polyprotein) peptide (amino acid sequence: RRGRVGRNPNQVGD) and HCV (1487–1500) peptide (amino acid sequence: RRGRTGRGRRGIYR) and WNV (1959–1972) peptide (amino acid sequence: RRGRIGRNPSGVGD) demonstrated a particular inhibitory effect on JEV NS3 helicase/NTPase activities. Among them, the HCV (1487–1500) peptide had the most potent inhibitory effect on JEV helicase activities, showing its great potential for targeted anti-JEV infection [59]. 

In addition, some inhibitors can ubiquitously inhibit the NS3 helicase/NTPase of flaviviruses. Ivermectin is a clinical drug used to treat human parasitic infectious diseases. Ivermectin was demonstrated to have the ability to inhibit the activity of flavivirus NS3 helicase, and its target is the single-stranded RNA (ssRNA) access site of the NS3 helicase domain, in which T408 and D409 are essential for ivermectin binding. Notably, ivermectin binds to the ssRNA access site of the NS3 helicase only in the presence of the substrate RNA. In vitro assays showed that ivermectin significantly inhibited the replication of several flaviviruses, including YFV, TBEV, and JEV. Because ivermectin is a safe drug, proven by clinical practice, the anti-JEV therapeutic effect of ivermectin has broad potential [60].

Other inhibitors can decrease JEV infectivity in vitro by inhibiting NS3 unwinding activity. Belladonna is a traditional botanical medicine with many clinical applications, such as anti-inflammation, anti-bacteria, and anti-oxidative stress, that has been widely used in clinical practice. The active components of belladonna are tropane alkaloids, which comprise atropine, scopolamine, and hyoscyamine. A hydroalcoholic belladonna (B200) formulation consisting of atropine and scopolamine showed its antiviral efficacy against JEV infection. Molecular docking analysis indicated that the target of atropine and scopolamine is the H288 residue of NS3, a crucial residue for NS3 RNA unwinding and ATPase activity [61]. Two heterocyclic organic compounds, AK-968/40733793 and AP-970/43253453, were found to significantly decrease JEV infectivity in vitro. In silico analysis showed that AK-968/40733793 and AP-970/43253453 targeted the unwinding channel contacting the DI and DII domains of NS3 helicase, but not the ATPase activity of NS3 helicase; it also demonstrated that H288, T451, and the R458 of NS3 are the key hydrogen bond donors for both AK-968/40733793 and AP-970/43253453 binding to NS3 [62]. 

### 4.5. Inhibitors Targeting NS5

Flaviviruses are vectored mainly by arthropods such as the Aedes and Culex mosquitos [63]. DENV, WNV, JEV, and YFV can be classified as established threats since they have affected the world’s health system over the years. Most of the published drug-development initiatives have used the active sites of NS5 as targets. Few JEV NS5-targeting drugs have been validated at the cellular or animal level practically, and several compounds that characteristically target the JEV NS5 remain in the early stages of in silico studies. Several compounds, including echinacoside, echinacin, rutin, cynaroside, quercetagetin 7-glucoside, and kaempferol-3-glucoside, have been shown to theoretically bind to JEV NS5 RdRp [64]. In addition, four bioflavonoid compounds, gedunin, nimbolide, ohchinin acetate, and kulactone, have been theoretically demonstrated to be capable of binding to the active site of JEV NS5 RdRp [65]. Nevertheless, due to the high conservation of NS5 in flaviviruses, NS5 inhibitors targeting flaviviruses such as DENV, WNV, and YFN can still be used as essential references for screening anti-JEV drugs.

#### 4.5.1. Nucleoside Analogue Inhibitors Targeting NS5 RdRp Active Site

Nucleoside inhibitors can compete with endogenous nucleoside triphosphates, and their assimilation into the new RNAs leads to either early termination or a lethal collection of mutations [66]. Several nucleotide inhibitors targeting flaviviruses’ NS5 active sites have been reported. A biphenyl acetic acid hit 3 could bind to a novel pocket in the palm subdomain of the RdRp; it is one derivative that has displayed antiviral activity for all four DENV serotypes at low micromolar concentrations in vitro [67]. Strong activity against ZIKV was observed for the *2′-C-methyluridine Aryloxyl Phosphoramidate ProTide’* triphosphate active metabolite, which is better incorporated by purified ZIKV NS5 polymerase [68]. Sofosbuvir became the first drug to replace interferon for HCV clinic treatment in 2014, which can bind to the active site of the HCV NS5B [69]. It was also confirmed to inhibit the purified WNV RdRp, and the S604T mutation within the catalytic site of RdRp affected the binding mode of sofosbuvir, suggesting that it might serve as a broad-spectrum inhibitor that targets NS5 of the *Flaviviridae* family viruses. Therefore, it is worth investigating whether sofosbuvir plays a role in JEV infection [70]. Remdesivir, a monophosphate prodrug of modified adenosine, has received widespread attention for its beneficial therapeutic efficacy in the treatment of COVID-19 [71]. It could also effectively inhibit the RdRp in various flaviviruses, including DENV3, tick-borne encephalitis virus (TBEV), JEV, WNV, and ZIKV. Thus, the effect of remdesivir in the treatment of JE is worthy of inspection [72]. BCX4430, a nucleoside analog targeting RdRp, displayed a broad spectrum of activity against various RNA viral pathogens, including YFV, JEV, DENV2, ZIKV, WNV, and TEBV [73,74,75]. Preliminary results from clinical trials of BCX4430 for yellow fever (YF) and coronavirus disease 2019 (COVID-19) have shown that it is safe and generally well tolerated in humans, and could reduce severe acute respiratory syndrome coronavirus 2 (SARS-CoV-2) viral load in the respiratory tract [75]. Consequently, it is important to investigate the targeted treatment of JEV infection with BCX4430 in more detail.

#### 4.5.2. Non-Nucleoside Inhibitors Targeting NS5 RdRp Active Site

One compound named TPB has been shown to potently inhibit ZIKV replication at sub-micromolar concentrations. Molecular docking analysis suggested that TPB binds to the catalytic active site of the RdRp and, therefore, likely blocks viral RNA synthesis [76].

#### 4.5.3. Allosteric Inhibitors Targeting NS5 RdRp

The first allosteric inhibitors, named compound 27 and compound 29, were found to work against DENV NS5 polymerase by targeting the N-pocket near the active site, thus interfering with the conformational transition, from initiation to elongation [77].

#### 4.5.4. Inhibitors Targeting NS5 MTase

The MTase inhibitor candidates are mainly designed to interact with the mRNA cap and SAM pockets. Derivatives containing a third phenyl ring linked to the original urea scaffold have been shown to inhibit DENV and ZIKV 2′-O-MTase activities. Two of these candidates have demonstrated the most efficient inhibition of MTase activity by binding to a proximal site of the SAM binding pocket, which is an allosteric site for MTase [78]. A novel flexible nucleoside analog of the FDA-approved nucleoside acyclovir that acts in the GTP pocket of DENV and ZIKV MTases has been discovered to inhibit viral replication in vitro [79]. N, N′-Carbazoyl-arylurea moiety inhibited ZIKV NS5 MTase by targeting the SAM binding site [80]. NSC 12155 could inhibit the replication of several flaviviruses, such as DENV2 and JEV, in vitro through the competitive inhibition of the cofactor SAM binding site to the MTase by hydrophobic interactions carried out in a dose-dependent manner [81].

### 4.6. Inhibitors Targeting JEV Lifecycle

The viral lifecycle appears to be complex, with multiple protein molecules involved at each step. However, the viral replication cycle is a single chain, and by inhibiting the function of either the viral or host’s element in that chain, a small step in viral replication can be affected, and thus viral replication can be inhibited.

#### 4.6.1. Virus Entry Inhibitors

The short peptides that are designed based on specific conserved sequences in the E protein of flaviviruses can theoretically inhibit virus entry by blocking the binding of the E protein to the receptor through competitive inhibition. The recombinant flavivirus E DIII proteins of WNV, DENV, and JEV were found to block the entry of JEV into BHK-21 cells and offer complete protection to JEV-infected mice [82]. A chemically synthesized peptide that was designed based on the loop3 (amino acid sequence: 362-ATSSANSKA-370) between βIII-βIV in the E DIII domain was found to effectively reduce JEV infectivity in vitro and in vivo, and acted in the adhesion stage of virus infection. Interestingly, it was only administered 2 h before JEV infection to protect animal models [83]. A short peptide (P5) that was designed based on the stem segment helix 2 of the E protein showed the remarkable inhibition of JEV infection in vitro and in vivo [84]. Therefore, envelope protein peptides represent a new direction in which to pursue potential therapeutic candidates for JEV infection. 

Although JEV-specific receptors have not been characterized to date, HS, a member of the GAGs, is considered an attachment receptor for JEV [85]. A recent study also demonstrated that the low-density lipoprotein receptor (LDLR) is a host factor required for JEV entry [86,87]. Inhibitors that directly or indirectly target HS or LDLR have an inhibitory effect on JEV infection. For example, bovine lactoferrin (bLF), an 80 kDa multifunctional iron-binding protein, could inhibit JEV cell entry by binding to HS and the LDLR-dependent JEV entry pathway by binding to LDLR [87]. Berbamine, a bis-benzylisoquinoline alkaloid isolated from berberis, which is often used in traditional Chinese medicine, could block TRPMLs (Ca^2+^ permeable non-selective cation channels in endosomes and lysosomes) to compromise the endolysosomal trafficking of LDLR and induce the secretion of LDLR via extracellular vesicles (EVs), thereby reducing the level of LDLR on the cell membrane and inhibiting the endocytosis of JEV. Animal experiments also showed that berbamine could significantly increase the survival rate of JEV-infected mice and reduce pathological changes in mouse brains [86].

Modern pharmacology studies show that many herbs and herbal ingredients can play a beneficial role in antiviral effects. *Isatis indigotica* is a herbal medicine used to treat virus infections, tumors, and inflammatory reactions, and its methanolic extracts, indigo, and indirubin, were found to be able to inhibit JEV infectivity by blocking JEV absorption and direct virucidal effects. Encouragingly, indirubin showed potent protective effects in a mouse model with a lethal JEV challenge [37]. Baicalein, belonging to the flavonoid group, also had a significant blocking effect on the adsorption of JEV and a strong anti-JEV infection effect [38].

#### 4.6.2. Inhibitors Targeting Post-Entry Stage

In the lifecycle of viruses, viral replication (genome synthesis) is the direct mode of viral proliferation, and the development of drugs that target the replication phase of viruses is a crucial and effective strategy for antiviral drug research. 

During the JEV replication stage, some host factors are involved in the viral RNA replication complex. The development of drugs that target these host factors could theoretically block virus replication to some extent. The valosin-containing protein (VCP)/p97, which is an abundant cellular ATPase with diverse cellular functions, was found to not only interact with the JEV NS5 and be an essential component of the virus replication complex, but was also found to govern viral nucleocapsid release from the clathrin-coated vesicle (CCV) [88]. CB-5083, an FDA-approved VCP inhibitor, was demonstrated to cause the significant inhibition of JEV RNA synthesis and viral titers, and to enhance the survival of JEV-infected mice, which suggests that it may play a role in the JEV RNA replication [88]. 

At the stage of JEV viral protein translation or posttranslational modification, some inhibitors exhibited significant anti-JEV activity. SCH16 demonstrated the robust inhibition of JEV infection at the translation stage of the viral protein in vitro. In addition, a survival experiment in JEV-infected mice showed that SCH 16 could significantly prolong the survival time of mice, and the survival protection rate for mice reached 100% [89]. *N*-nonyl-deoxynojirimycin (*N*N-DNJ), a 9-carbon alkyl iminosugar derivative, is an α-glucosidase I and/or α-glucosidase II inhibitor in ER. *N*N-DNJ exerted an anti-JEV effect by reducing the synthesis and secretion of JEV virus glycoprotein and disrupting the correct folding of the viral glycoprotein. In a mouse model of the lethal challenge posed by JEV infection, *N*N-DNJ significantly improved the survival rate of mice [90]. Therefore, targeting the translation of JEV viral proteins is a new proposal in the search for anti-JEV drugs, but it is essential to consider that these drugs may also interfere with the synthesis of host cell proteins.

Several inhibitors have been demonstrated to block JEV infection by disrupting the viral assembly or release process. A compound that was modified based on specific groups of pyridobenzothiazolones was found to significantly inhibit the infectivity of various flaviviruses in vitro, including JEV, DENV, and YFV, and exerted its effect on YFN by causing the release of viruses that were unable to complete a second round of infection; this suggests that its target may be a viral functional protein that is responsible for virus packaging [91]. Pentoxifylline, a methylxanthine derivative that has been used for treating human vascular diseases [92], was demonstrated to have a strong inhibitory effect on JEV infection in vitro and in vivo by inhibiting the assembly and/or release of the virion [93].

Although a vast number of anti-JEV infection agents, such as the AR-12 derivatives P12–23 and P12–34 [94], have been shown to function at a particular stage of the viral lifecycle, their specific targets remain unclear, and their antiviral molecular mechanisms require in-depth investigation. Such agents not discussed here are listed in Table 1.

### 4.7. Inhibitors Targeting Innate Immune and Inflammatory Responses

Innate immune response and inflammatory activation are recognized as key obstacles in the process of virus invasion. Various drugs can inhibit the replication of the virus by regulating the innate immune response and inflammatory response, and are able to critically protect the host, particularly in regard to the CNS.

On the one hand, the IFN-mediated antiviral response mainly includes type I and type IIIFN responses [122]. To our knowledge, TRLs play an essential role in the IFN-mediated innate host antiviral response. An embryonated chick infection experiment showed that scopolamine hydrobromide could significantly inhibit the viral load of JEV in the chorioallantoic membrane (CAM) and brain by remolding the TLR and IFN signaling pathways [100]. Certain drugs may augment IFN-I-mediated antiviral responses in order to limit JEV replication. Platelet cytokine PF4, upon binding to its receptor CXCR3, inhibits the IFN-α antiviral response that is induced by virus infection and efficiently promotes the replication of JEV and DENV2 [111]. As a CXCR3 antagonist, AMG487, at a concentration of 2.5 μM, could inhibit PF4-mediated JEV replication in mouse microglial BV2 cells and restore IFN-α secretion in DENV2-infected monocytes. Furthermore, AMG487 treatment in mice reduced JEV infection and increased mice survival [111]. Additionally, some agents can induce IFN-II antiviral response. Aloe emodin and chrysophanol, the methanol extracts of *Rheum palmatum*, were demonstrated to inhibit JEV replication by activating gamma-activated sequence (GAS)-driven genes to indirectly initiate IFN-γ -triggered host innate immune responses against JEV infection in vitro [39]. Another in vitro study demonstrated that aloe emodin could inhibit JEV replication by activating the IFN-stimulated response element (ISRE) and GAS-driven cis-reporting systems associated with the activation of type I and II IFN responses, and the up-regulation of the expression of IFN-stimulated genes [120]. 

On the other hand, several antiviral candidates have been found to protect the CNS and prolong the survival time of JEV-infected animals by modulating the inflammatory mediators released by microglial activation and inducing neuronal death. Some drugs are effective in anti-JE therapy by inhibiting microglia activation and inflammatory regulation in vivo. Fenofibrate, which is used as a hypolipidemic drug, is an agonist of peroxisome proliferator-activated receptor-α(PPARα), which could abrogate JEV-mediated microglial activation and the release of inflammatory mediators; it also was found to have a protective effect on neuroblastoma in vitro and in vivo [116,123,124,125]. Rosmarinic acid (RA), a phenolic compound found in various Labiatae herbs, could effectively reduce the replication of JEV in the brain and the levels of secondary inflammatory factors such as IL-12, TNF-α, IFN-γ, MCP-1, and IL-6 that result from the activation of microglia in vivo, suggesting that it might be a strong candidate for the treatment of JE in the future [114]. 

There are also drugs that, in addition to their inflammation-modulating effects, have impressive anti-apoptotic effects. 2-(2-Methyl-quinoline-4ylamino)-N-(2-chlorophenyl)-acetamide (PP2) is an anilidoquinoline derivative with excellent protective effects in JEV-infected mice, which acts through the regulation of apoptotic molecules such as Bcl2, Bax, and caspase-3 [118]. Minocycline, a small semisynthetic tertracycline, was found to abrogate microglial activation and the induction of pro-inflammatory cytokines in a JEV-infected animal model, and prevent JEV-induced neuronal death by decreasing caspase-3 activity [112]. Moreover, there are drugs with a protective impact on the blood–brain barrier (BBB). For instance, treatment with minocycline could protect the BBB of JEV-infected mice by inhibiting leukocyte migration into the brain by up-regulating the adhesion molecules and decreasing the activity of matrix metalloproteinase (MMP)-9, which initiates the degradation of the BBB [113].

The modulation of the inflammatory response and cell death through the inhibition of microglia activation is an essential mechanism of such drugs that act against JEV infection. Apart from the several high-potential drugs mentioned above that have significant effects regarding immune and inflammatory modulation or anti-JEV infection, there are also other drugs that, at the in vitro and/or in vivo level, provide protection to hosts due to JEV infection to a certain extent; these are listed in Table 1.

## 5. Outlook

Currently, more and more drugs have been reported to be able to inhibit JEV infection. Nonetheless, there are still no clinically approved anti-JE drugs to date. Apart from minocycline, a second-generation antibiotic that has been demonstrated to have a neuroprotective effect on JE in several animal model experiments, no meaningful results have been summarized from the clinical trials of the minocycline treatment of JE due to the low number of JE infection cases [126]. Nonetheless, it remains of concern to continually explore the clinical benefit of minocycline against JEV infection. 

In the *Flaviviridae* family, NS proteins are essential viral elements for the replication of viruses and are crucial targets and hot spots for antiviral drug design. Although no drugs characteristically target NS proteins for the clinical treatment of JEV infection, several inhibitors targeting HCV NSs have been widely used in clinical settings with beneficial outcomes [127]. For example, the NS3/4A protease inhibitor pibrentasvir, NS5A inhibitor glecaprevir, NS5B polymerase inhibitor sofosbuvir, NS5A inhibitor velpatasvir and NS3/4A protease inhibitor voxilaprevir, all of which have shown promising effects in the clinical treatment of anti-HCV infection, may subsequently provide insights that can be used in the research of novel anti-JEV drugs. 

Additionally, SCH16, an N-methylisatin-β-thiosemicarbazone derivative, showed promising and intense anti-JEV infection effects at concentrations below the nanomolar level, and significant protection against JEV infection in JEV-infected mice. Furthermore, another study demonstrated that SCH16, combined with ribavirin and mycophenolic acid at the cellular level, had a synergistic effect against JEV infection [106]. Due to this, it is necessary to investigate further the anti-JEV infection mechanism of SCH16 and its potential clinical applications.

## 6. Final Conclusions

JEV is a mosquito-transmitted flavivirus, and current research focuses on targets for JEV antivirals, mainly the virus itself and factors related to the host. The development of antiviral drugs should be based on a thorough understanding of the lifecycle of JEV. It is, therefore, important to carefully select drug targets to ensure effective antiviral activity, broad-spectrum properties, and minimal toxicity to the host. This review has highlighted several approaches to the design of anti-JEV infection drugs, as follows:

(1) Computer drug design is a convenient and rapid means of drug development and can save costs and time in drug screening. It can identify the amino acid residues in the target protein that interacts with the drug when the precise structure of the viral target protein is known. However, this is still at the theoretical design and analysis stage, and the actual situation needs to be validated by cellular and animal assays. Combined with the results of computer theoretical analyses, the targeted mutation of specific amino acids in target proteins may be an effective means to explore the exact molecular information regarding the interaction between drugs and their targets.
(2) High-throughput drug screening (HTS) at the cellular level is currently an increasingly used tool for antiviral drug development. Several compound libraries contain hundreds of thousands of compounds, and with automated cell manipulation techniques and convenient viral labeling and detection tools, candidate drugs can be identified efficiently and rapidly. In those compound libraries, natural compounds are deserving of attention since they show few side effects and are also easily accessible from natural sources, such as berbamine, rosmarinic acid, arctigenin, and indirubin, all of which could effectively inhibit JEV infection in animal models.
(3) The synthesis of derivatives based on the modification of effective leading compounds. For example, the excellent anti-JEV infection effect shown in vitro and in vivo by the anilidoquinoline analogue PP2 suggests that modifications based on clinically used drugs with potential antiviral effects are an effective means of developing novel anti-JEV drugs.
(4) Drug repurposing, which has become a popular and convenient drug discovery method, intends to uncover new therapeutic properties of old drugs. Minocycline, temoporfin, niclosamide, nitazoxanide, berbamine and fenofibrate are all in clinical use for the treatment of different diseases, but they have also been shown to have potential effects against JEV infection in vivo. Together with these drugs’ safety in humans, they are promising for the clinical treatment of JE.
(5) In-depth analysis of the functions and sequences of the E proteins and NSs proteins of flaviviruses in order to locate the conserved sequences that affect the functions of these viral proteins, as well as the recombinant expression or chemical synthesis of antiviral short peptide inhibitors based on these sequences, is a theoretically feasible and convenient method for antiviral drug development. However, due to the complex structure of E or NSs, these short peptides of viral origin may not completely inhibit the function of E proteins or NSs, and their immunogenicity is of concern.

Besides the methods mentioned above, the development of drugs that specifically cut off the transmission route through mosquito bites is not only a novel idea for JEV antiviral research, but is also a practical and feasible approach. Retnla, which encodes resistin-like molecule-α (RELMα), is an antimicrobial protein on host skin; flaviviruses, such as DENV2 and ZIKV, can suppress the expression of RELMα, which leads to the robust growth of acetophenone-producing bacteria on host skin, resulting in increased acetophenone levels on the skin; this is a volatile compound that is produced by skin-resident bacteria and is attractive to mosquitoes. Vitamin A derivatives such as isotretinoin have been shown to induce RELMa expression, thereby ultimately reducing acetophenone levels on the skin and thus reducing mosquito bites [128]. Therefore, the dietary administration of vitamin A derivatives can contribute to the prevention of JEV infection, especially in high-JE-endemic regions.

Knowledge of JEV has increased dramatically via in-depth studies of its virology, host factors, and innate immune and inflammatory response, which give a boost to the continuous development of candidate drugs in JE treatment. Therefore, further efforts are required in order to research and develop JEV antiviral drugs.

## Figures and Tables

**Table 1:** Antivirals for JEV infection.

Classification of Virus Infection Stages	Target/Mechanism	Classification of Antivirals	Name of Antivirals	Study Stages/Experimental Model	
				In Vitro	In Vivo
Pre-infection	Extracellular virucidal activity	Lipase secreted by bacterium	CbAE-1 and CbAE-2 [42]	Vero cell	-
		Plant-derived antiviral protein	Griffithsin [41]	BHK-21 cell	BALB/c mice
		Inorganic compounds	POM-12 [40]	Vero cell	-
		Natural compounds	Aloe emodin and chrysophanol [39]	BHK-21 cell	-
			Baicalein and quercetin [38]	Vero cell	-
			Luteolin [95]	A549 cell	-
			Indirubin, indigo [37]	BHK-21 cell	BALB/c mice
Entry stage	E	Neutralizing antibodies	2F2 and 2H4 [43]	Vero cell	BALB/c mice
		Nanomedicines	Cur-CQDs [47]	BHK-21 cell	-
		GAG mimics	Shrimp HS [50]	BHK-21 cell	-
			Heparin [48]	BHK-21 cell	-
		Antiviral peptides	P3 [45]	BHK-21 cell	-
		Small-molecule inhibitors	BP34610 [44]	BHK-21 cell	-
	Viral particle or E	GAG mimics	PI-88 [49]	BHK-21 cell	C57Bl/6
	Block JEV absorption	Natural compounds	Indirubin, indigo [37]	BHK-21 cell	BALB/c mice
		Plant-derived antiviral protein	Griffithsin [41]	BHK-21 cell	BALB/c mice
		Nanomedicines	NSQc [96]	BHK-21 cell; human neuronal HTB-11 cell	C57BL/6 mice
	Decrease in LDLR level at the plasma membrane	Licensed drug; natural compounds	Berbamine [86]	A549 cell	BALB/c mice
	HS; LDLR	Animal derived natural anti-microbial protein	bLF [87]	BHK-21 cell; HS-expressed CHO-K1 and HS-deficient CHO-pgsA745 cell	-
	Inhibit virus binding	Virus-derived peptides	Recombinant flavivirus DIIIproteins [82]	BHK-21 cell	BALB/c mice
			P5 [84]	BHK-21 cell	C57BL/6 mice deficient in type I and II IFN receptors
			Loop3 [83]	BHK-21 cell	BALB/c mice
Post-entry stage	NS2B/NS3 protease	Small-molecule inhibitors	SK-12 [56]	Vero cell	-
		FDA-approved food additive	Erythrosin B [55]	A549 cell	-
		Licensed drugs	Temoporfin, niclosamide and nitazoxanide [54]	A549 cell	Balb/c mice and A129 mice (anti-ZIKV of temoporfin)
		Natural compounds	Andrographolide [57]	Protease inhibitory assay	-
			4-hydroxypanduratin A [53]	In silico	-
			Abscisic acid and aloe-emodin [58]	Protease inhibition assay	-
	NS3 helicase/NTPase	Virus-derived peptides	HCV(1487–1500), WNV(1959–1572), JEV(1962–1975) [59]	Biochemistry assay	-
		Licensed drugs	Atropine and scopolamine [61]	SHSY-5Y/CHME3 cell	-
			Ivermectin [60]	Vero E6 cell	-
	NS3 helicase	Small-molecule inhibitors	AK-968/40733793 and AP-970/43253453 [62]	BHK-21 cell	-
		Natural compounds	5,11-Dihydroindolo [3, 2-β] carbazole and perlolyrine [97]	In silico	-
	NS3 NTPase	Nucleotide Analogues	Ring-Expanded Nucleoside (REN) Analogues [98]	ATPase and helicase assay	-
	NS5 MTase	Small-molecule inhibitors	NSC 12155 [81]	A549 cell	-
	NS5 RdRp	Natural compounds	Echinacoside, echinacin, rutin, cynaroside, quercetagetin 7-glucoside, and kaempferol-3-glucoside [64]	In silico	-
			Gedunin, nimbolide, ohchinin acetate, and kulactone [65]		-
		Licensed drugs	Remdesivir [72]	Recombinant flaviviral polymerases assay	-
		Nucleoside analogs	BCX4430 [75]	Vero cell	-
			2′-C-methyl-cytidine 217 or 2′-C-MeC, 7-deaza-7-fluoro-2′-C-methyl-adenosine [99]	Vero cell	-
	NS5	Licensed drugs	Scopolamine [100]	Embryonated chick	-
		Natural compounds	5,11-Dihydroindolo [3, 2-β] carbazole and perlolyrine [97]	In silico	-
	Target histone deacetylase 6 (HDAC6) –Hsp90-NS5-RNA replication axis	Small-molecule inhibitors	Tubacin [101]	Human cerebellar medulloblastoma TE671 cell	-
	Inhibit viral RNA synthesis	Plant-derived antiviral protein	PAP [102]	BHK-21 cell	BALB/c mice
		Small-molecule inhibitor	FGIN-1-27 (N,N-dihexyl-2-(4-fluorophenyl)indole-3-acetamide) [103]	BHK-21 cell	-
			P12–23 and P12–34 [94]	Human lung carcinoma A549 cell	P12–34 against DENV in Stat1^−/−^ mice
		Licensed drugs	CB-5083 [88]	HeLa cell	C57BL/6 mice
			Cilnidipine and niclosamide [103]	BHK-21 cell	-
		Synthetic compounds	HAAS-AV3026 and HAAS-AV3027 [104]	BHK-21 cell; Vero cell	-
	Suppress virus replication combined with modest inhibition of virus entry	Licensed drugs	Lonafarnib [105]	Huh7 cell	-
	Disrupt viral translation	Small-molecule inhibitors	SCH 16 [89,106]	Porcine stable kidney (PS) cell	Swiss albino mice [89]
	Disrupt viral posttranslational modification	Small-molecule inhibitors	*N*N-DNJ [90]	BHK-21 cell	ICR mice
	Disrupt virus assembly	Synthetic compounds	Compound 16 [91]	Vero cell	-
	Disrupt virus assembly and/or release	Licensed drugs	Pentoxifylline [93]	PS cell	Swiss albino mice
	Disrupt post-entry stage	Natural compounds	Luteolin [95]	A549 cells	-
		Synthetic compounds	4-(5-phenyl-1,2,4-oxadiazol-3-yl)-N-(pyridin-3-ylmethyl)aniline(5d) [107]	BHK-21 cell	-
	Bind to JEV frameshift site RNA	Natural compounds	Kaempferol and daidzin [108]	BHK-21 cell	-
	Target the ubiquitin proteasome system-related genes	Licensed drugs	Bortezomib [109]	-	BALB/c mice
Innate immune and inflammatory responses	Remould the TLR and IFN signaling Pathways	Licensed drugs	Scopolamine [100]	-	Embryonated chick
	Induce IFN signaling responses	Natural compounds	Abscisic acid and aloe-emodin [58]	Protease inhibition assay	-
			Aloe-emodin [110]	Human TE-671 medulloblastoma cell and HL-CZpromonocyte cell	-
	Activate PF4-CXCR3-IFN axis	Small-molecule inhibitors	AMG487 [111]	BV2 cell; THP-1 cell	BALB/c mice
	Trigger host innate immune responses by activating GAS-driven promoter	Natural compounds	Aloe emodin and chrysophanol [39]	BHK-21 cell	-
	Regulate Inflammatory response	Licensed drugs	Minocycline [112,113]	-	BALB/c mice
	Modulate the release of proinflammatory cytokines and chemokines	Natural compounds	Rosmarinic acid [114]	-	BALB/c mice
	Antiviral; neuroprotective; anti-inflammatory; immunomodulatory	Antimicrobial peptides (AMPs)	Tilapia hepcidin 1–5 [115]	BHK-21 cell	C3H/HeN mice
	Neuroprotection	Licensed drugs	Atropine and scopolamine [61]	SHSY-5Y/CHME3 cell	-
			Fenofibrate [116]	BV-2 microglial cell	BALB/c mice
			Memantine [117]	-	C57BL/6 mice
		Synthetic compounds	2-(2-Methyl-quinoline-4ylamino)-N-(2-chlorophenyl)-acetamide(PP2) [118]	Mouse neuroblastoma (Neuro-2a) cell	BALB/c mice
	Inhibition of microglial activation	Natural compounds	Arctigenin [119]	Neuro-2a cell	BALB/c mice
	Anti-oxidative	Synthetic compounds	Compounds 1b and 1f [120]	Neuro-2a cell	BALB/c mice
	Anti-oxidative; dysregulation of UPS	Licensed drugs	Curcumin [121]	Neuro-2a cell	-

## Data Availability

Not applicable.

## Abbreviations

**Table:**

Acronym	Full Name
JEV	Japanese encephalitis virus
DENV	Dengue virus
ZIKV	Zika virus
YFV	Yellow fever virus
SINV	Sindbis virus
WNV	West Nile virus
HCV	Hepatitis C virus
TBEV	Tick-borne encephalitis virus
SARS-CoV-2	Severe acute respiratory syndrome coronavirus 2
JE	Japanese encephalitis
COVID-19	Coronavirus disease 2019
YF	Yellow fever
GI	Genotypes I
GII	Genotypes II
GIII	Genotypes III
GIV	Genotypes IV
GV	Genotypes V
ORF	Open-reading-frame
UTRs	Untranslated regions
C	Capsid protein
PrM	Precursor membrane protein
M	Membrane protein
E	Envelope protein
FP	Fusion peptide
DI	DomainI
DII	DomainII
DIII	DomainIII
NS	Nonstructural protein
SAM	S-adenosyl methyltransferase
MTase	Methyltransferase
RdRp	RNA-dependent RNA polymerase
ssRNA	Single-stranded RNA
IFN	Interferon
TNF	Tumor necrosis factor
PRRs	Pattern recognition receptors
TLRs	Toll-like receptors
RIG-I	Retinoic acid-inducible gene I
RLRs	Retinoic acid-inducible gene I (RIG-I)-like receptor
IL	Interleukin
ROS	Reactive oxygen species
Bcl2	B-cell lymphoma 2 protein
GAS	Gamma-activated sequence
ISRE	IFN-stimulated response element
CNS	Central nervous system
BBB	Blood-brain barrier
ER	Endoplasmic reticulum
TGN	Trans-Golgi network
POMs	Polyoxometalates
GRFT	Griffithsin
CQDs	Carbon quantum dots
CUR	Curcumin
*N*N-DNJ	*N*-nonyl-deoxynojirimycin
B200	A hydroalcoholic belladonna formulation
TPB	3-chloro-N-[({4-[4-(2-thienylcarbonyl)-1-piperazinyl]phenyl}amino)carbonothioyl]-1-benzothiophene-2-carboxamide
PP2	2-(2-Methyl-quinoline-4ylamino)-N-(2-chlorophenyl)-acetamide
mAbs	Monoclonal antibodies
CbAE	Chromobacterium antiviral effector
bLF	Bovine lactoferrin
PAP	Plant-derived antiviral protein
AMPs	Antimicrobial peptides
RA	Rosmarinic acid
UPS	Ubiquitin-proteasome system
PF4	Platelet factor 4
HDAC6	Histone deacetylase 6
Hsp90	Heat shock protein 90
RELMα	Retnla encodes resistin-like molecule-α
MMP	Matrix metalloproteinase
MCP-1	Monocyte chemoattractant protein-1
GAGs	Glycosaminoglycans
HS	Heparan sulfate
LDLR	Low-density lipoprotein receptor
TRPMLs	Ca^2+^ permeable non-selective cation channels in endosomes and Lysosomes
PPARα	Peroxisome proliferator-activated receptor-α
EVs	Extracellular vesicles
VCP	Valosin-containing protein
CCV	Clathrin-coated vesicle
CAM	Chorioallantoic membrane
PTSA	Protein thermal shift assay
HTS	High-throughput drug screening
